# Exploring Mechanisms and Therapeutic Strategies for Metformin Hydrochloride-Induced Diarrhea and Gastrointestinal Intolerance: A Bibliometric Analysis

**DOI:** 10.3390/medsci14060621

**Published:** 2026-09-30

**Authors:** Angelo Elias Meri Junior, Emily Caroline Cardoso Batista, Adrila David Meri, Ana Cristina La Guardia Custódio Pereira, Fabiangelo Moura Carlos, Vinicius Silva Belo, Ana Julia Pereira Santinho Gomes, Cristina Sanches, Sukyung Woo, André Oliveira Baldoni, Tiago Silveira Gontijo

**Affiliations:** 1 Dona Lindu Center-West Campus, Federal University of São João del-Rei, 400 Sebastião Gonçalves Coelho Street, Chanadour, Divinópolis 35501-296, MG, Brazil; cardoso.batista.emily@hotmail.com (E.C.C.B.); anaclaguardia@gmail.com (A.C.L.G.C.P.); fabiangelomc@gmail.com (F.M.C.); viniciusbelo@ufsj.edu.br (V.S.B.); ajpsant@ufsj.edu.br (A.J.P.S.G.); csanches@ufsj.edu.br (C.S.); andrebaldoni@ufsj.edu.br (A.O.B.);; 2 Santa Mônica Divinópolis Hospital Hapvida NotreDame Intermédica, Divinópolis 35502-562, MG, Brazil; adrila.meri@hapvida.com.br; 3 Department Pharmaceutical Sciences, School of Pharmacy & Pharmaceutical Sciences, University at Buffalo, The States University of New York (SUNY), Buffalo, NY 14214, USA; skwoo@buffalo.edu

**Keywords:** diabetes mellitus, type 2, drug-related side effects and adverse reactions, gastrointestinal microbiome, bibliometrics, scientific publication indicators

## Abstract

**Background:** Metformin hydrochloride (MET) gastrointestinal adverse effects (GAEs) are associated with intestinal accumulation, gut microbiota alterations, transporter saturation, and increased serotonin and lactate levels. This study mapped the scientific literature indexed in Web of Science (WoS) and Scopus, with MET-induced gastrointestinal intolerance and diarrhea as the primary research focus, concentrating on mechanisms and strategies to mitigate GAEs. **Methods:** This bibliometric analysis followed the PRISMA and PICO guidelines. WoS and Scopus were searched using terms derived from DeCS/MeSH descriptors. Two independent reviewers performed study selection. Data were analyzed using Bibliometrix (biblioshiny) in RStudio for performance analysis, scientific mapping, and collaboration networks. **Results:** A total of 6998 articles were identified, resulting in the inclusion of 153 studies (116 original articles and 37 reviews), distributed across 117 journals. The annual growth rate was 4.74%, with a peak in productivity between 2022 and 2025, and an average of 45.01 citations per document. GAEs constituted the most investigated area (28.1%), followed by molecular mechanisms (11.8%) and associated comorbidities (10.5%). The United States led scientific production (*n* = 37), followed by China (*n* = 18) and India (*n* = 17). **Conclusions:** Despite advances in understanding mechanisms, interventional studies and therapeutic strategies remain underrepresented. Strengthening international collaboration and participation from low- and middle-income countries is essential to develop more effective and equitable approaches.

## 1. Introduction

According to the International Diabetes Federation, 588.7 million people worldwide had diabetes mellitus (DM) in 2024 [1]. DM comprises heterogeneous conditions, among which type 1 diabetes mellitus (DM1) results from autoimmune destruction of pancreatic β cells, leading to absolute or near-absolute insulin deficiency and typically manifesting in younger individuals [2,3]. Although clinically significant, DM1 accounts for a relatively small proportion of cases. In contrast, type 2 diabetes mellitus (DM2) represents approximately 90–95% of all DM cases and is therefore the predominant form worldwide. Its occurrence is strongly associated with modifiable and demographic factors such as obesity, unhealthy dietary patterns, physical inactivity, and population aging, with diagnosis occurring more frequently after the age of 40. Pathophysiologically, DM2 is characterized by peripheral insulin resistance, involving reduced glucose uptake by muscle and adipose tissue and increased hepatic glucose production [2,3].

The treatment of individuals in the early stages of DM2 involves lifestyle changes, particularly improvements in eating habits and physical activity. When these changes are insufficient to control blood glucose, the continuous use of medicines should be initiated [2,3]. Metformin hydrochloride (MET) is considered the first-line treatment for DM2 [4]. Its mechanism of action involves the inhibition of gluconeogenesis, reduced glucose absorption in the intestine, and increased glucose reuptake by muscles. Consequently, the drug is classified as an antihyperglycemic agent [5].

Adverse effects associated with MET use are predominantly gastrointestinal intolerance, with diarrhea representing the most significant barrier to patient adherence [6]. This adverse event is particularly common during the initial phase of treatment and is more frequently observed with immediate-release formulations [7]. Although extended-release (XR) formulations have been developed to mitigate gastrointestinal intolerance, evidence indicates that diarrhea, while less frequent, can still occur with these formulations [8].

MET is an orally administered drug belonging to the biguanide class, absorbed primarily in the small intestine [5]. It is further classified as a Class III drug under the Biopharmaceutics Classification System (BCS) by virtue of its high solubility and low passive permeability [9]. However, despite its low passive transcellular permeability, MET absorption is largely mediated by active uptake transporters, specifically, the organic cation transporters expressed in enterocytes. Consequently, its bioavailability (approximately 40 to 60%) is more strongly influenced by transporter activity than by passive diffusion. It is known that the greater the accumulation of MET in the intestine, resulting from its rapid release, the higher the likelihood of adverse gastrointestinal events [10,11].

In the gastrointestinal tract, MET is transported across the membrane via organic cation transporters (OCTs), primarily OCT1 and OCT3, which mediate its uptake by enterocytes. Additionally, other transporters, such as the plasma membrane monoamine transporter (PMAT), may also contribute to intestinal absorption. Conversely, transporters such as OCT2 are primarily involved in renal elimination, while multidrug and toxin extrusion (MATE1 and MATE2) are associated with hepatic and renal efflux processes. MET intolerance may occur when intestinal uptake transporters are compromised, whether due to the action of other drugs, genetic polymorphisms, or saturation, leading to the accumulation of MET in the intestinal lumen [10,12,13,14]. It is worth noting that, following oral administration, MET can reach intraluminal concentrations estimated between 10 and 100 mM, which are substantially higher than the reported IC50 values for OCT1-mediated transport processes. This corroborates the likelihood of transporter saturation under physiological conditions [15].

According to Dawed et al. (2019), the PMAT, also responsible for MET transport, may undergo genetic variation, resulting in the accumulation of MET in the lumen and, consequently, an increased risk of adverse gastrointestinal effects [16]. Furthermore, MET and serotonin share transport pathways due to their cationic nature. Serotonin is transported primarily via influx mechanisms involving the serotonin transporter (SERT) and organic cation transporters. Thus, competition between MET and serotonin for these transporters may lead to increased intraluminal serotonin levels, which has been associated with symptoms such as nausea, vomiting, and diarrhea [10,15].

Another mechanism related to MET-induced gastrointestinal intolerance involves increased lactate production within the intestinal lumen. MET inhibits mitochondrial complex I in enterocytes, leading to a transition from oxidative phosphorylation to anaerobic glycolysis, which elevates intracellular and luminal lactate levels. This is the same mitochondrial mechanism responsible for the rare, yet severe, adverse effect of lactic acidosis. This metabolic alteration may contribute to an imbalance in the intestinal microbiota, as elevated lactate levels can affect bacterial metabolic pathways, including the inhibition of enzymes such as glycerol-3-phosphate dehydrogenase in certain species. This mechanistic link, by connecting local gastrointestinal toxicity (microbiota imbalance) to systemic pharmacokinetic concerns (risk of lactic acidosis), reinforces the importance of minimizing intestinal exposure to the drug through formulation strategies [6,10,17,18,19]. The imbalance in the intestinal microbiota can also be attributed to the toxic effects of MET on certain bacterial species that produce folic acid, such as those belonging to the genera *Lactobacillus* and *Bifidobacterium*. Consequently, symptoms such as cramps, abdominal pain, and diarrhea may arise due to this imbalance [20,21,22].

Bibliometric analysis is a tool used to understand the historical trajectory of research within any academic field. By providing a quantitative review of the literature and analyzing citation frequencies, this approach reveals key patterns, including the most prominent scientific investigations, organizations, institutions, and international collaborations, as well as the most influential authors. Furthermore, it is capable of demonstrating the association between the impact and growth of a specific topic, identifying knowledge trends, key themes, study types, and current ideas and concepts [23]. Although various bibliometric studies have explored different aspects of research regarding MET, there is a notable scarcity of studies specifically focused on the gastrointestinal intolerance and diarrhea associated with its use [24,25,26,27]. Previous analyses have not comprehensively addressed the underlying mechanisms or potential therapeutic strategies for mitigating these adverse effects. In this regard, this gap is particularly significant, given that gastrointestinal intolerance constitutes one of the primary factors limiting treatment adherence.

This study aims to explore and map scientific literature indexed in Web of Science (WoS) and Scopus, using bibliometric analysis, regarding MET-induced gastrointestinal intolerance and diarrhea, specifically focusing on studies where these outcomes were the primary subject, with particular emphasis on underlying mechanisms and reported therapeutic strategies. Furthermore, this study seeks to identify and characterize key thematic areas within the literature, including topics such as transporters, gut microbiota, and metabolic pathways, to provide a structured overview of the field. By identifying research trends, influential studies, and emerging themes, this analysis aims to provide a comprehensive understanding of the current state of knowledge and inform future research in this area.

## 2. Materials and Methods

The research question and conceptual scope of this study were initially structured using the PICO framework: population of interest—patients using MET; intervention—studies investigating mechanisms, contributing factors, or therapeutic approaches related to MET-induced gastrointestinal adverse effects; comparison—not applicable or comparison with standard MET use without modifications; and outcome—gastrointestinal intolerance and/or diarrhea. The PICO framework was used to guide the research question and define the conceptual scope of this investigation; however, its components were not directly applied as restrictive criteria for database searches or study eligibility. The search strategy and eligibility criteria were developed in accordance with the objectives and scope of the bibliometric analysis. This bibliometric analysis was conducted and reported in accordance with the PRISMA guidelines [28]. A set of quantitative descriptive data and scientific information analysis techniques were used, which were regrouped into two main categories: performance analysis and scientific mapping [29].

As inclusion criteria for peer-reviewed studies, those that investigated/explained the possible mechanisms related to gastrointestinal intolerance or diarrhea associated with the use of MET, and studies that proposed new strategies to reduce or eliminate these adverse effects were selected. Documents were excluded if they mentioned MET only in the introduction or discussion, the use of MET in neoplasms, or case reports addressing lactic acidosis.

Studies were considered to address gastrointestinal intolerance or diarrhea as a primary outcome when this outcome was explicitly stated as the main objective, hypothesis, or focus of the title/abstract, or when it represented the central finding discussed in the results. Studies in which gastrointestinal intolerance/diarrhea was reported merely as a secondary or incidental safety outcome, without constituting a stated study objective, were excluded, consistent with the exclusion criterion for studies that mentioned MET only in the introduction or discussion.

To reduce subjectivity, the central finding criterion was applied only when the abstract or results explicitly reported gastrointestinal intolerance/diarrhea as a measured outcome (e.g., incidence, severity, or discontinuation rate) rather than as an incidental observation. Eligibility decisions were made using a dual-reviewer strategy. Discrepancies were resolved through joint discussion, with arbitration by a third reviewer when consensus could not be reached. Interviewer agreement was assessed using Cohen’s Kappa coefficient (see Section 2.1).

### 2.1. Data Collection and Preparation

The WoS and Scopus databases were selected to retrieve information relevant to publications exploring the theme of this study. These databases were chosen due to their international recognition as multidisciplinary platforms, offering broad coverage and the ability to search and classify results by specific parameters [30,31]. PubMed was not included in the search strategy. Although it is a key reference source in the health sciences, this database provides a more limited set of exportable metadata compared to WoS and Scopus, which restricts its suitability for the bibliometric indicators calculated in Bibliometrix/biblioshiny [32,33]. For this reason, WoS and Scopus were prioritized as the primary data sources for this study.

The Health Sciences Descriptors and Medical Subject Headings (DeCS/MeSH) controlled vocabulary was used during the search strategy construction phase to identify relevant terms and synonyms (e.g., “diarrhea”, “gastrointestinal side effects”). These terms were then combined with additional free-text keywords and applied as free-text searches in the “Topic” (WoS) and “TITLE-ABS-KEY” (Scopus) fields, as neither database supports direct searching of MeSH indexing in the same way as PubMed. In the WoS database, the following search strategy was applied: (metformin) AND (“gastrointestinal intolerance” OR diarrhea OR “gastrointestinal adverse effect*” OR “gastrointestinal side effect*” OR “digestive intolerance” OR “abdominal discomfort” OR “abdominal pain” OR “gastrointestinal tract”). We used “Topic” as a search field, where you search for terms in the title, abstract, keyword plus, and author keywords. The initial search returned 907 results. Subsequently, the filters “Article” and “Review Article” were applied to select only original and review articles, excluding 29 non-peer-reviewed publications. This resulted in 878 results as of 29 October 2025.

In the Scopus database, the search strategy used was: (metformin) AND (“gastrointestinal intolerance” OR diarrhea OR “gastrointestinal adverse effect*” OR “gastrointestinal side effect*” OR “digestive intolerance” OR “abdominal discomfort” OR “abdominal pain” OR “gastrointestinal tract”). We use “TITLE-ABS-KEY” as a search field, where you search for terms in the title, abstract, and keyword. The initial search returned 6091 results. Using the filters “Article” and “Review,” 480 non-peer-reviewed publications were excluded, leaving 5611 results as of 29 October 2025. The complete, verbatim search strings, filters, and search dates for both databases are provided in Supplementary Table S1.

Editorials, abstracts, case reports, notes, news articles, guidelines, and corrections were excluded. Additionally, studies conducted on animal species other than rodents were excluded due to limited translational relevance to human physiology. There were no restrictions regarding language or period.

To contextualize the present study and highlight its originality, we explore previously conducted bibliometric analyses related to MET, with a focus on their research scope and search strategies (Table 1). This comparison enables the identification of differences in thematic focus and methodological approaches, thereby substantiating the rationale for the present investigation.

The search and selection of articles were conducted by two authors (A.E.M.J. and E.C.C.B.) simultaneously and independently. The discrepancies were assessed by a third evaluator. A total of 6998 records were initially identified in WoS (*n* = 907) and Scopus (*n* = 6091). After applying document type filters, which excluded 509 records, 6489 results remained and were exported as a BibTeX file to Bibliometrix (biblioshiny) [32] in RStudio [34]. Duplicate articles (*n* = 668) were then removed, leaving 5821 records. These 5821 articles were reviewed using Microsoft Excel^®^, based on their titles and abstracts, to determine their eligibility for inclusion in this study. The selected articles were then read in full, and disagreements were resolved through joint review and discussion, leading to their inclusion or exclusion.

Furthermore, the Kappa index was calculated to assess inter-observer agreement using IBM^®^ SPSS^®^ statistical software (version 31), resulting in 0.50 in the first stage and 0.90 in the second stage of study selection. These values can be interpreted according to Altman et al. (1991), indicating a moderate and almost perfect of agreement in the two selection stages, respectively [35].

The search strategy intentionally prioritized sensitivity over specificity, including broader terms like “gastrointestinal tract” to minimize the risk of missing relevant studies, given the heterogeneous terminology used in pharmacological and clinical literature to describe gastrointestinal outcomes associated with MET. This high-sensitivity approach explains the large number of initially retrieved records. The subsequent screening of titles/abstracts and full texts, conducted independently by two reviewers, with arbitration by a third reviewer and assessment of Kappa agreement, was designed to compensate for this lower specificity.

### 2.2. Data Analysis

Data from the selected publications were extracted from WoS and Scopus to perform the bibliometric analysis. These data included keywords, author names, country of origin, institutions, journals of publication, publication dates, citations, g-index, and h-index.

Based on the search, the retrieved data were exported as a file to Bibliometrix (biblioshiny) in RStudio (version 4.5.2) to generate information related to the growth and trends of publications, international and author collaborations, citations, keywords, and frequency related to the study theme.

The data were also used to create network visualization maps, in which parameters such as countries and authors were represented by circles that varied in size depending on the weight of the item and were differentiated by color. The lines between the items represented the links, and their thickness indicated the strength of each link.

The studies were classified into the following thematic areas based on their primary research focus, determined by analyzing the title, abstract, and full text: gastrointestinal effects, pharmaceutical formulations and delivery systems, pharmacokinetics and pharmacodynamics, gut microbiota, clinical efficacy and glycemic control, treatment adherence and tolerability, molecular mechanisms and metabolic pathways, comorbidities and associated diseases, combination therapy and pharmacological combinations, safety and non-gastrointestinal adverse effects, and specific populations. Each study was assigned to a single predominant thematic category to avoid double-counting. When a study addressed more than one theme, the classification was based on the outcome or mechanism most emphasized in the title, objectives, and conclusions. Discrepancies in classification were resolved through assessment by the two reviewers, with arbitration by a third author when necessary.

### 2.3. Declaration of Generative AI Use

A generative AI tool (ChatGPT, GPT-5.6 Luna, OpenAI) was used exclusively to assist in generating and designing the graphical abstract summarizing this study’s findings. No generative AI or LLM tool was used for the generation, analysis, or interpretation of the manuscript’s text, data, or scientific content. The authors reviewed and took full responsibility for the accuracy and scientific content of the final graphical abstract.

## 3. Results

**Figure 1:**
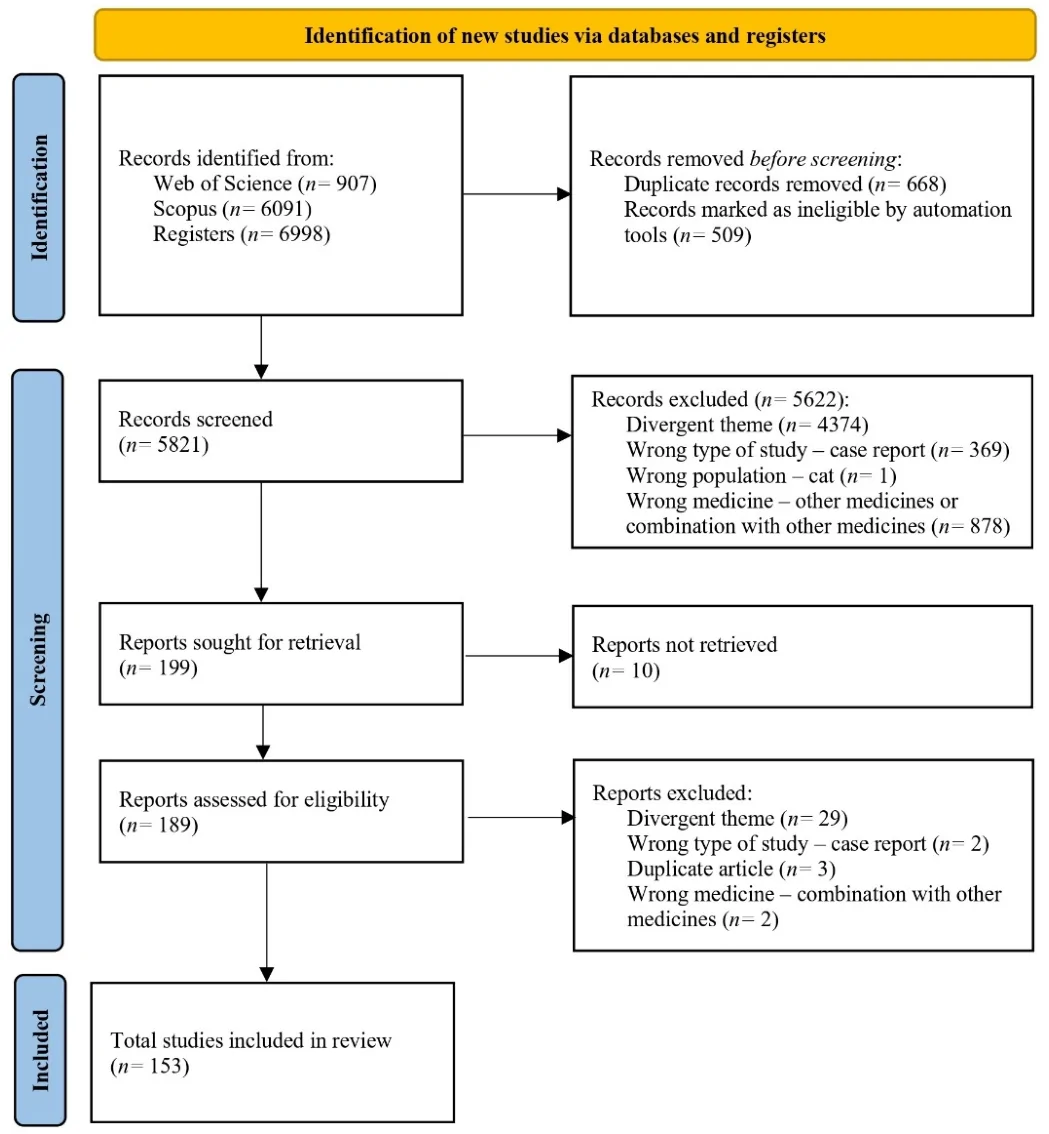
PRISMA flowchart of the selection steps for articles included in this review.

A total of 6998 articles were found in the WoS (*n* = 907) and Scopus (*n* = 6091) databases. After applying filters (*n* = 509) and removing duplicates (*n* = 668), 5821 articles were analyzed according to the inclusion and exclusion criteria. Of these, 5668 were excluded, resulting in 153 articles included in this review (Figure 1).

The substantially higher number of records retrieved from Scopus compared to WoS is consistent with Scopus’s broader journal coverage and more inclusive indexing policies. This is a well-documented difference between the two databases [30]. A total of 117 sources (scientific journals) were found. The annual growth rate of publications was 4.74%. The average citations per document was 45.01, and a total of 439 unique references were identified across the dataset. A total of 2056 Keywords Plus^®^ and 346 author keywords were identified. Seven hundred and eighty-four (*n* = 784) authors were identified, 7 of which had single-authored documents. In terms of author collaboration, the average number of co-authors per document was 5.89, and 1.31% of the articles had international co-authorships. The document types included 116 articles and 37 reviews.

**Figure 2:**
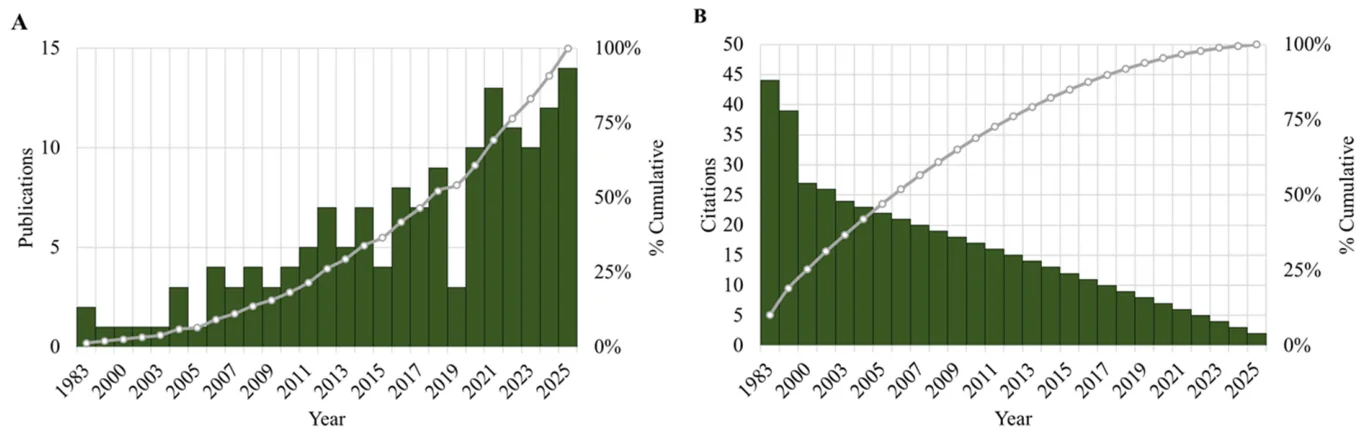
Annual scientific production (**A**) and average annual citations (**B**) between 1983 and 2025. Notes: The gray line represents the cumulative percentage.

Although duplicate articles were initially identified and removed using the Bibliometrix package (biblioshiny) in RStudio, additional duplicate records were detected during the peer review process. This occurred due to minor variations in metadata across databases (differences in punctuation, hyphenation, or formatting of titles and author names), that were not recognized by the automated tool. These additional duplicates were removed prior to the bibliometric analyses reported here. Therefore, all analyses were performed using the fully deduplicated final dataset (*n* = 153). Figure 2 illustrates the temporal evolution of the scientific literature on diarrhea and gastrointestinal intolerance induced by MET.

The annual number of publications remained relatively low from 1983 until the early 2000s, followed by a gradual increase after 2010. A more pronounced growth is shown in the last 10 years, reaching its highest annual productivity between 2022 and 2025. This steady upward trajectory is evidenced by the cumulative percentage (gray line), indicating possible continued expansion of the research field over time. In contrast, the average annual number of citations (Figure 2B) was higher in the early years and progressively decreased over time, likely reflecting the longer period available for citation accumulation. Additionally, the cumulative citation curve (gray line) rises rapidly during the early years and gradually approaches saturation, which refers to the association of a large proportion of total citations with older publications.

Table 2 summarizes the ten most influential authors in this field, including their institutions, ORCIDs, h-indexes, and the articles they published, along with publication year, DOI, and citation counts. Among them, Ewan Pearson stands out as the leading contributor, with an h-index of 80 and five publications directly related to the topic, encompassing highly cited studies on molecular mechanisms, transporters (OCT and SERT), and MET formulations. Yu Wang (*n* = 4) and Ilze Elbere (*n* = 3) also demonstrate notable contributions, particularly in research on extended-release formulations and gut microbiota, respectively. Additionally, authors such as Vivian Fonseca, Janusz Gumprecht, and Jānis Kloviņš have produced highly cited works, further reinforcing their relevance within this research domain.

Table 3 presents the distribution of the 153 studies included in this bibliometric analysis, categorized by the identified thematic areas. The gastrointestinal effects of MET constituted the primary area of investigation, accounting for 43 studies (28.1%). Overall, it is observed that scientific literature has prioritized aspects related to the gastrointestinal tolerability of the drug. Conversely, areas such as molecular mechanisms (*n* = 18; 11.8%) and associated comorbidities (*n* = 16; 10.5%) reflect a growing interest in understanding the systemic effects and expanded clinical applications of MET.

**Figure 3:**
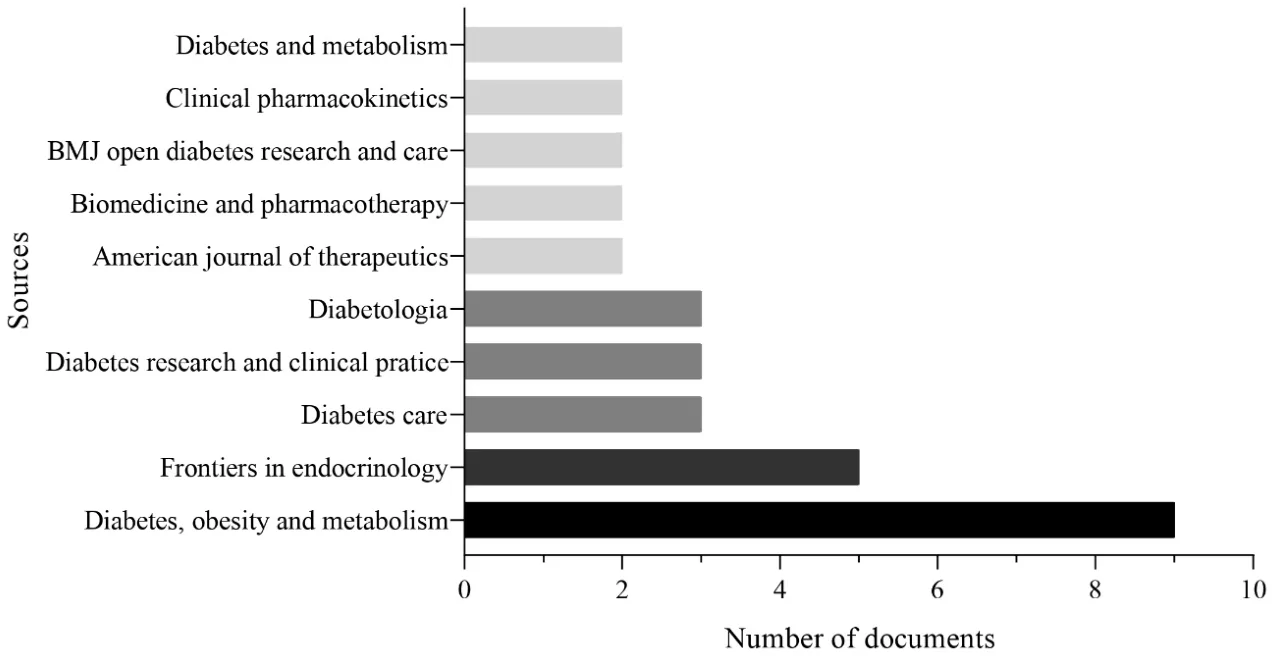
The 10 journals that published the largest number of articles used in this bibliometric analysis.

Figure 3 presents the distribution of the number of documents by source (journals) included in this bibliometric analysis. It is noteworthy that the journal “Diabetes, Obesity and Metabolism” accounts for the highest number of publications (*n* = 9; 5.9%). Following this, “Frontiers in Endocrinology” occupies the second position (*n* = 5; 3.3%). An intermediate group of journals, including “Diabetes Care”, “Diabetes Research and Clinical Practice”, and “Diabetologia”, present similar contributions (*n* = 3; 2.0%).

**Figure 4:**
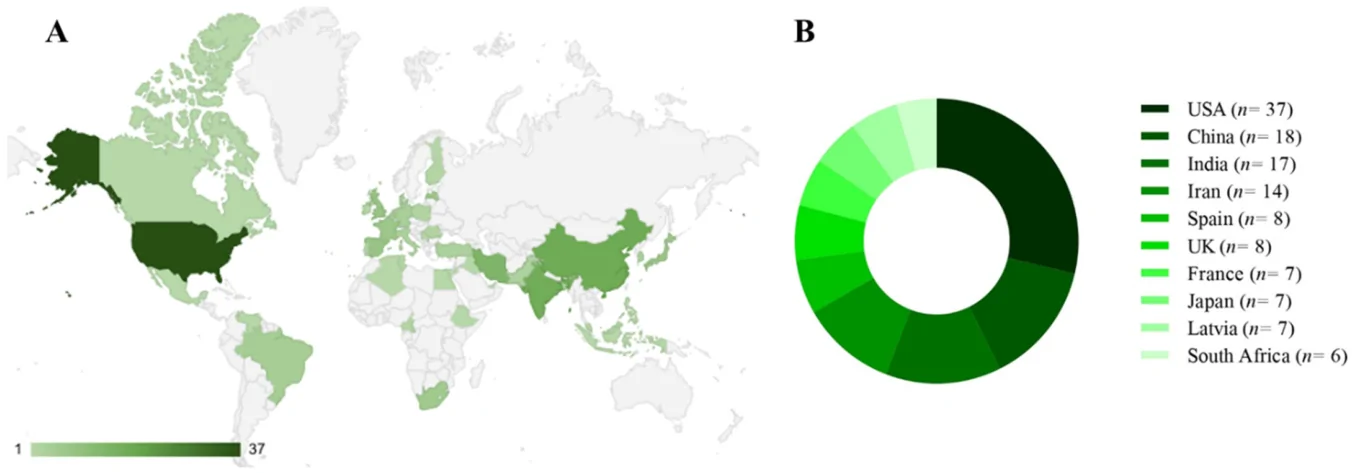
Geographic distribution of publications (**A**) and top ten countries’ contributions (**B**) in this bibliometric analysis.

Figure 4 presents the geographic distribution of the included publications. A global dispersion of studies is observed, with a higher concentration of publications in North America, Europe, and Asia. Regions such as South America and Africa show a lower density of publications indexed in WoS and Scopus (Figure 4A). This pattern may reflect a combination of factors, including differences in database coverage, language of publication, research funding, and scientific infrastructure, rather than necessarily indicating lower scientific engagement with the topic in these regions. The United States (*n* = 37) stands out as the country with the highest number of publications, followed by China (*n* = 18) and India (*n* = 17).

**Figure 5:**
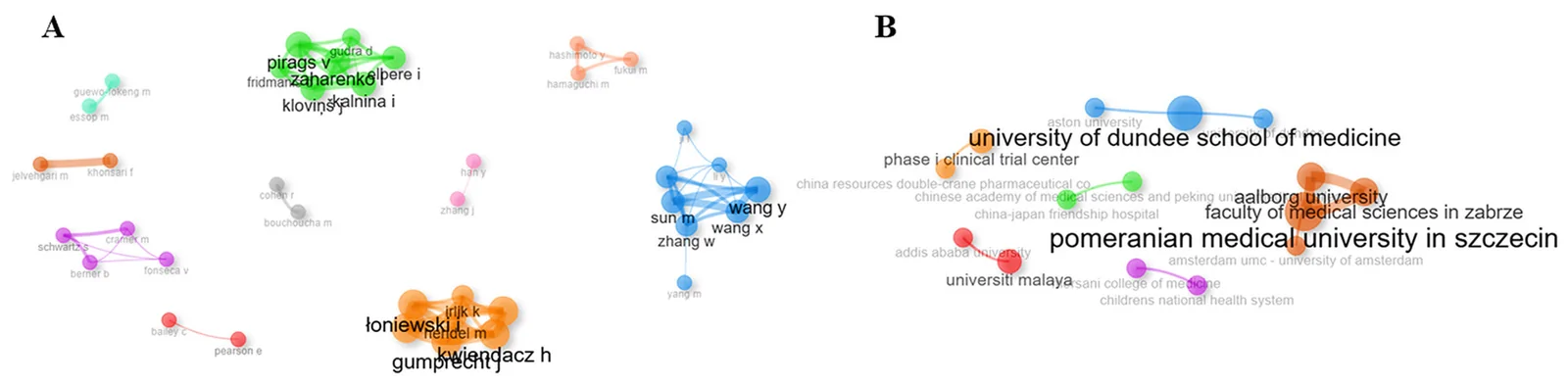
Author (**A**) and institutional (**B**) collaboration networks from articles included in this bibliometric analysis.

Figure 5 displays the author’s collaboration networks. The author network (Figure 5A) consists of ten clusters, characterized predominantly by small, disconnected groups and low intermediation. Wei Zhang exhibits the highest betweenness value (6.0), a metric representing the capacity to act as a “bridge” within the network, while many authors (e.g., Ewan Pearson and Clifford Bailey) have a value of zero. In contrast, the institutional network (Figure 5B) reveals six clusters. It combines high intermediation (betweenness = 1.0) with the highest PageRank (0.097), a metric that underscores the role of these institutions as hubs of collaboration, while the Pomeranian Medical University in Szczecin exhibits the highest institutional betweenness (2000). Additionally, several institutions display maximum closeness (1000), such as Universiti Malaya and Addis Ababa University, indicating potentially high structural accessibility. In this regard, both networks exhibit distinct collaborative patterns: authors are distributed across fragmented groups, whereas institutions form more centralized and hierarchical structures.

According to Figure 5A, Ewan Pearson and Zhang I. were considered the main authors in the network within their respective clusters, but no collaboration occurred between the two authors. The same observation can be made in Figure 5B, where the main universities in the network within their respective clusters were the University of Dundee (Scotland), Nanjing Medical University (China), Baylor College of Medicine (United States), and Emory University (United States), but no collaboration occurred between them.

**Figure 6:**
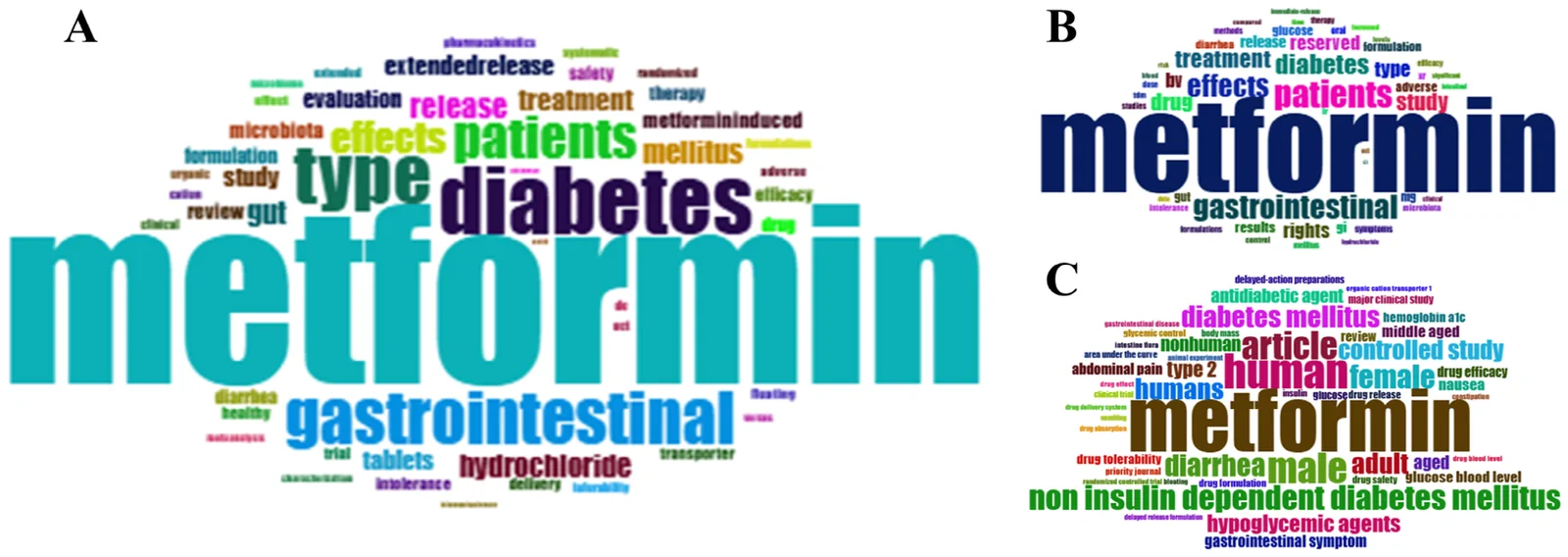
Most frequent keywords from titles (**A**), abstracts (**B**), and Keyword Plus^®^ (**C**) in this bibliometric analysis.

Figure 6 presents the most frequently occurring keywords related to the title, abstract, and Keywords Plus^®^. The keyword “metformin” was the most frequently identified across the three analyzed categories (title, *n* = 131; abstract, *n* = 889; Keywords Plus^®^, *n* = 214). Furthermore, other frequently occurring keywords include “diabetes” (*n* = 50) and “type” (*n* = 44)—Figure 6A (title); “patients” (*n* = 257) and “gastrointestinal” (*n* = 227)—Figure 6B (abstract); and “human” (*n* = 118) and “male” (*n* = 104)—Figure 6C (Keywords Plus^®^).

## 4. Discussion

To the best of our knowledge, this is the first bibliometric study focused specifically on MET-induced gastrointestinal intolerance and diarrhea, with an emphasis on underlying mechanisms and therapeutic strategies. Our results reveal progressive growth in scientific output over time, with a sharp increase in the last ten years and a peak in productivity between 2022 and 2025. Conversely, older publications have accumulated higher average citation counts, reflecting their foundational role in the field. A wide dispersion of studies across 117 scientific journals was also observed, with prominence given to certain specialized outlets that concentrated a larger number of publications. Additionally, there is a concentration of scientific output in high-income countries, primarily the United States, China, and India, consistent with limited international collaboration among authors, although more structured institutional networks do exist. In parallel, a small group of highly influential authors has contributed to the development of the field, specifically in areas related to pharmacogenetics, drug transporters, and formulation strategies. In the analysis of thematic distribution, there was a predominance of topics such as gastrointestinal tolerability, molecular mechanisms, and associated comorbidities. Keywords indicate a strong association with clinical and gastrointestinal aspects, reinforcing the relevance of this adverse effect within the context of MET therapy. Our findings offer important insights into evolution, structure, and current directions of research regarding MET-induced gastrointestinal intolerance.

It is worth noting that, as this is a bibliometric analysis, our study aims to characterize publication trends, thematic clusters, and collaboration patterns, rather than establish causal biological mechanisms or clinical efficacy. The mechanistic and pharmacological information discussed below reflects the content reported in the primary studies included in our dataset and is presented to provide context for the thematic mapping, not as new evidence generated by the present analysis.

The steady increase in scientific output observed in our findings over recent years can likely be attributed to multiple factors. First, the global rise in the prevalence of DM2 has intensified the clinical use of MET and, consequently, the significance of its gastrointestinal adverse effects [1,50,57]. Concurrently, advancements in fields such as pharmacogenetics, drug transport mechanisms, and the gut microbiota have provided a better understanding of MET’s mechanisms of action and its side effects [10,58,59]. The growing interest in personalized medicine may also have contributed to the expansion of research, particularly in studies investigating individual variability in drug response and tolerability [60,61,62,63,64,65].

Concurrently, reviews regarding MET have supported the use of genomics, metabolomics, and the microbiome to personalize the selection and dosage of medications used in the treatment of DM2 [66,67]. Pharmacomicrobiomics is considered an applied strategy for predicting efficacy and gastrointestinal side effects, utilizing the MET as the primary model [68]. A study conducted by Naja et al. (2023) [64], involving 119 MET users from the Qatar Biobank, identified the presence of distinct metabolic profiles between good and poor responders. Those considered good responders demonstrated elevated levels of sphingomyelins, acylcholines, and glutathione metabolites, whereas poor responders exhibited higher levels of glucose metabolism metabolites and gut microbiota products [64]. In this context, it is considered that genetics explains part of the variability; therefore, combining metabolomics and microbiota may enhance the individual prediction of efficacy in the use of MET [65].

The thematic analysis revealed that gastrointestinal effects constituted the most frequently investigated area, representing 28.1% of the included studies, followed by molecular mechanisms and metabolic pathways (11.8%) and by comorbidities and associated conditions (10.5%). This distribution indicates a strong emphasis on understanding the clinical manifestations and biological mechanisms underlying MET intolerance, consistent with the potential and growing interest in broader metabolic and systemic contexts associated with its use [69].

The higher average number of citations observed in older publications reflects the so-called “citation time effect”. In this phenomenon, older studies have more time to accumulate citations [70]. In this regard, they frequently represent seminal studies that established the foundations of current knowledge, particularly concerning topics such as pharmacokinetic profiles and interactions with intestinal transporters, specifically OCT and SERT, of MET. More recent publications, although greater in number, have not demonstrated comparable citation levels. This fact likely does not reflect a lesser scientific relevance of these studies, but rather a shorter period of exposure within the literature [71].

Various studies have demonstrated limitations when evaluating citations of recently published articles. Adams et al. (2005) indicate that annual citations tend to peak around the third year [71]. Furthermore, they point out a strong correlation between early citations (years 1 to 2) and long-term citations (years 3 to 10) due to the fact that more recent works have not yet had sufficient time to accumulate a volume of citations comparable to older ones [71]. Bormann (2013) argues that a minimum period of approximately three years is required for citation indicators to be meaningful [72]. The appeal of an article diminishes over time, giving rise to the “aging effect”. Consequently, more recent articles face comparative disadvantages in impact analyses based on accumulated citations [73].

The predominance of publications originating from high-income countries, such as the United States, China, and India, points to potential inequalities regarding funding, infrastructure, and scientific capacity [74,75,76]. According to the International Diabetes Federation, these countries exhibit a high prevalence of DM2, with approximately 38.5, 148.0, and 89.8 million people living with the disease in 2024, respectively [1]. Furthermore, they possess well-established investments in biomedical research, which may facilitate the conduct of large-scale studies and foster greater international visibility [77,78]. Conversely, global disparities in scientific output are evident, marked by the lower participation of regions such as Africa and South America, thereby highlighting the need for more inclusive initiatives. The promotion of research in these regions should be grounded in and guided by potential variations in environmental factors, genetics, and healthcare systems, all of which may influence MET tolerability [75,79,80].

The analysis of scientific journals revealed a wide dispersion of the retrieved articles, distributed across 117 sources, which may demonstrate the multidisciplinary nature of the topic. Despite this variation, the journal “Diabetes, Obesity and Metabolism” accounted for the highest number of publications (*n* = 9; 5.9%), followed by “Frontiers in Endocrinology” (*n* = 5; 3.3%). These are likely the leading publication venues within this field of knowledge, distinguished by their clinical and translational focus while also highlighting the diversity of the involved disciplines, including pharmacology and endocrinology [81,82,83]. Additionally, traditional journals in the field, such as “Diabetes Care”, “Diabetes Research and Clinical Practice”, and “Diabetologia”, also made significant contributions (*n* = 3; 2.0% each), thereby reinforcing the clinical profile of the scholarly output [84,85]. This observed pattern suggests that scientific production is likely directed toward specialized journals, further reinforcing the clinical and translational profile of the topic. Concurrently, the distribution across multiple journals underscores the integration and expansion of research into various disciplines, including pharmacology, microbiology, and pharmaceutical technology [86,87].

The analysis of the collaboration networks in the present study revealed a pattern characterized by fragmentation among authors and greater structuring at the institutional level. This finding may possibly indicate that researchers tend to work in relatively isolated groups, despite institutions acting as organizing hubs for research [88,89]. This fragmented behavior may reduce knowledge sharing, as well as opportunities for high-impact multicenter studies [89,90,91]. In this context, strengthening interdisciplinary and international collaboration can significantly aid in the advancement of knowledge, particularly in complex fields such as gut microbiota and pharmacogenetics [92,93,94].

An analysis of the most prolific authors provides complementary insights into the thematic orientation and methodological profile of the studies included in this bibliometric analysis. The contributions of these authors reflect a heterogeneous, yet complementary, set of research approaches. Our findings range from mechanistic investigations to the development of pharmaceutical formulations and clinical evaluations. Of the 153 articles analyzed, 116 are original articles (75.8%) and 37 are reviews (24.2%), indicating a predominance of experimental and observational investigations in understanding the mechanisms related to MET-induced gastrointestinal intolerance.

A more robust analysis of studies associated with the most prolific authors reveals the existence of diverse research domains within the field. Studies conducted by authors such as Ewan Pearson and colleagues have focused primarily on pharmacogenetic mechanisms and drug transporters, specifically OCT and SERT, demonstrating genetic variability in the occurrence of gastrointestinal intolerance caused by MET [10,36,37,38]. Conversely, authors such as Ilze Elbere and Jānis Kloviņš focused their analyses on the gut microbiota, corroborating how alterations in microbial composition can influence both the emergence of gastrointestinal effects and therapeutic efficacy during the use of MET [44,45,46].

Another important field of study identified among the most prolific authors involves the development and evaluation of pharmaceutical formulations. Researchers such as Fatemeh Khonsari and Mitra Jelvehgari have contributed significantly through studies involving the development of modified-release formulations or mucoadhesive/buccoadhesive systems, with the aim of enhancing the tolerability of MET and reducing associated gastrointestinal intolerance. These formulation-based approaches represent a practical strategy for improving patient adherence to pharmacotherapy by modifying or enhancing drug release kinetics and reducing intestinal accumulation [53,54,55].

Furthermore, among the retrieved studies, those conducted by Wang and colleagues stand out; they evaluated the bioequivalence and safety of different extended-release MET formulations, demonstrating the potential of these strategies to improve treatment tolerability [40,42,43]. In parallel, Janusz Gumprecht has contributed more recent investigations exploring therapeutic interventions aimed at reducing gastrointestinal intolerance, including the use of probiotics in patients intolerant to MET [50,51,52].

This bibliometric analysis also encompassed studies investigating strategies aimed at increasing drug permeability or optimizing its pharmacokinetic performance, a trend that likely reflects a growing effort to overcome the intrinsic limitations of MET, given its classification as a Class III drug under the BCS [95]. In this context, modified-release formulations (e.g., XR) have been proposed as a strategy to mitigate gastrointestinal intolerance. From a pharmacokinetic perspective, XR formulations shift the absorption of MET to more distal regions of the gastrointestinal tract, thereby reducing the high intraluminal concentrations in the proximal intestine, where transporter-mediated uptake is most pronounced [6,7,10,19]. This results in a lower maximum plasma concentration (Cmax) while maintaining overall systemic exposure (AUC), which may contribute to reducing the saturation of intestinal transporters and improving tolerability, as reported in the included literature. Furthermore, delayed-release formulations have been developed to target distal intestinal regions even more specifically, thereby reinforcing this strategy of modulating local drug exposure while simultaneously maintaining glycemic control [96,97]. Taken together, our results indicate that the contributions of the most prolific authors are not restricted to the volume of publications, but are strongly integrated with the key translational challenges of the research field, particularly those related to the development of new pharmaceutical formulations, drug delivery, and modulation of the gut microbiota [98,99,100,101,102].

Overall, the thematic distribution observed in this review suggests a concentration of research efforts on clinical manifestations and mechanistic aspects of MET intolerance, highlighting the central role of gastrointestinal effects and molecular pathways in the current scientific landscape. This shift may represent the maturation of the field and corroborates the importance of multidisciplinary approaches in developing strategies to reduce gastrointestinal intolerance associated with MET use, integrating pharmacology, microbiology, and pharmaceutical technology [6,58,69,103].

The keyword analysis reinforces the main research themes identified throughout this bibliometric analysis. The predominance of terms such as “metformin”, “diabetes”, “patients”, and “gastrointestinal” indicates a strong clinical orientation within the literature, highlighting the importance of MET-induced gastrointestinal intolerance in the context of DM2 treatment [6,50,51]. Additionally, the presence of keywords associated with biological mechanisms, such as microbiota and transporters, suggests a growing interest in identifying factors that modulate drug tolerability and therapeutic response [68,104,105,106,107].

Given that this is a bibliometric review focused on MET-induced gastrointestinal intolerance, clinical terms and those related to the digestive system were the most frequent keywords (Figure 5). However, a detailed thematic analysis of the included studies reveals the potential integration of these terms into broader conceptual clusters, including molecular transport mechanisms (OCT, PMAT, SERT), gut microbiota dysbiosis, pharmacokinetic determinants, and formulation-related strategies (Table 3). This distribution suggests that the research field has likely been steadily expanding, ranging from descriptive clinical reports to mechanistic and translational investigations, although interventional and therapeutic studies remain comparatively limited.

We acknowledge the non-inclusion of the PubMed database as a limitation. Although considered a relevant source in the health sciences for the topic at hand, this database provides a more restricted set of exportable metadata compared to WoS and Scopus [33], which limits some of the analyses, based on citations, collaborations, and keywords, conducted in this bibliometric study. Consequently, our results should be interpreted as representative of the literature indexed in WoS and Scopus, rather than the entirety of global scientific output on the subject. Furthermore, we highlight that this exclusion may have resulted in the systematic omission of clinical guidance publications indexed predominantly or exclusively in PubMed, particularly case reports, clinical trials, and pharmacology-focused journals with limited overlap regarding WoS and Scopus coverage. This represents a potential source of selection bias in our mapping.

Additionally, our eligibility criteria required gastrointestinal intolerance/diarrhea be the primary focus of this study, rather than a secondary or incidental safety outcome. Consequently, the total number of included studies (*n* = 153) should not be interpreted as representative of the entire body of literature regarding MET-related GAEs, but specifically of the literature in which these outcomes were a central research focus. Studies reporting such GAEs merely as secondary safety findings were not covered by this mapping and may offer additional information not reflected in this study.

Overall, this study highlights important patterns in the scientific development of research regarding MET-induced gastrointestinal intolerance, while simultaneously revealing gaps that warrant further investigation. The fragmentation observed among authors, combined with the limited participation of low- and middle-income countries, suggests a need for globally integrated and representative research efforts. Furthermore, although significant advances have been made in understanding the mechanisms underlying gastrointestinal adverse effects, our findings suggest that research efforts remain largely focused on mechanistic and descriptive approaches, with comparatively fewer studies addressing therapeutic strategies to mitigate these effects. To foster a more detailed understanding of this topic, and ultimately, to improve patient adherence and therapeutic outcomes, strategies such as strengthening international collaboration and expanding research in underrepresented regions are essential.

## 5. Conclusions

This bibliometric analysis reveals a progressive expansion of research on MET-induced gastrointestinal intolerance, marked by an increase in publications over the past ten years, as well as a predominance of studies related to clinical gastrointestinal effects. Thematic mapping also highlights growing interest in molecular mechanisms, interactions with the gut microbiota, and other comorbidities, although interventional studies and therapeutic strategies remain comparatively underrepresented. Furthermore, scientific output is concentrated in high-income countries and characterized by fragmented authorship networks, indicating structural gaps in global collaboration. Thus, our findings suggest that future research should prioritize investigating potential targeted interventions, such as transporter-related mechanisms, microbiota modulation, approaches to address increased permeability, and optimized pharmaceutical formulations, as promising avenues for improving MET tolerability and developing new therapeutic strategies. Furthermore, future bibliometric or systematic reviews that expand database coverage (e.g., PubMed and OpenAlex) would further enhance the comprehensiveness of this mapping within the research field.

## Figures and Tables

**Table 1:** Characteristics of previous bibliometric studies on metformin hydrochloride regarding the databases and search strategies applied.

Reference	Title	Aims	Database	Search Strategy
Pan et al., 2024/China [24]	Knowledge mapping of metformin use on cancers: a bibliometric analysis (2013–2023)	To identify the major contributing factors and delineate the current research status within this domain, thereby providing valuable insights for clinical professionals and researchers interested in this field	WoS	(TS = (Metformin OR Glucophage OR N, N-1 dimethylbiguanide OR N-1,1-dimethylbiguanide) AND TS = (Cancers OR Neoplasms OR Tumors OR Carcinomas))
Tang et al., 2024/China [25]	A bibliometric analysis of global research trends in polycystic ovary syndrome and metformin	To understand current development trends and research foci related to the use of metformin hydrochloride in polycystic ovary syndrome	WoS	“topic = Polycystic ovary syndrome AND topic = metformin”
Shu et al., 2023/ China [26]	Bibliometrics and visual analysis of metformin and gut microbiota from 2012 to 2022: A systematic review	To conduct a bibliometric analysis of publications regarding metformin and the gut microbiota over the past 11 years (2012–2022), identify key contributors (including authors, countries, and institutions) and the current state of research, as well as elucidate the research focus and trends in this field	WoS	Theme = (metformin) AND Theme = (“Gastrointestinal Microbiome” or “Gastrointestinal Microbiomes” OR “Gut Microbiome” OR “Gut Microbiomes” OR “Gut Microflora” OR “Gut Microbiota” OR “Gut Microbiotas” OR “Gastrointestinal Flora” OR “Gut Flora” OR “Gastrointestinal Microbiota” OR “Gastrointestinal Microbiotas” OR “Gastrointestinal Microbial Community” OR “Gastrointestinal Microbial Communities” OR “Gastrointestinal Microflora” OR “Gastric Microbiome” OR “Gastric Microbiomes” OR “Intestinal Microbiome” OR “Intestinal Microbiomes” OR “Intestinal Microbiota” OR “Intestinal Microbiotas” OR “Intestinal Microflora” OR “Intestinal Flora” OR “Enteric Bacteria”)
Song et al., 2021/China [27]	A bibliometrics analysis of metformin development from 1980 to 2019	To build up essential, reliable, and thorough information on metformin in the last 40 years since its early emergence	WoS	topic = (“Metformin”)

**Table 2:** Top 10 authors: institutions, ORCID, h-index, publications, year, citations, and DOI.

Author	Institution	ORCID	H-Index	Published Articles	Year	Number of Citations	DOI
Ewan Pearson	University of Dundee, United Kingdom	https://orcid.org/0000-0001-9237-8585	80	Pharmacokinetics of metformin in patients with gastrointestinal intolerance [36]	2018	42	https://doi.org/10.1111/dom.13264
				Organic cation transporter 1 variants and gastrointestinal side effects of metformin in patients with type 2 diabetes [37]	2016	117	https://doi.org/10.1111/dme.13040
				Metformin and the gastrointestinal tract [10]	2016	581	https://doi.org/10.1007/s00125-015-3844-9
				Effect of serotonin transporter 5httlpr polymorphism on gastrointestinal intolerance to metformin a GODARTS study [38]	2016	41	https://doi.org/10.2337/dc16-0706
				Adherence in patients transferred from immediate release metformin to a sustained release formulation a population based study [39]	2009	60	https://doi.org/10.1111/j.1463-1326.2008.00973.x
Yu Wang	Capital Medical University, China	https://orcid.org/0000-0002-0278-0405	18	Bioequivalence and safety assessment of two formulations of metformin hydrochloride sustained release tablets Yuantang SR and Glucophage XR under fed conditions in healthy Chinese adult subjects an open label two way crossover sequence randomized phase I clinical trial [40]	2022	8	https://doi.org/10.1007/s40268-021-00377-w
				Bioequivalence and safety evaluation of two preparations of metformin hydrochloride sustained release tablets boke and Glucophage XR in healthy Chinese volunteers a randomized phase I clinical trial [41]	2022	7	https://doi.org/10.1080/07853890.2022.2125574
				Pharmacokinetic bioequivalence and safety assessment of two metformin hydrochloride tablet formulations using a phase I randomized open two period two crossover single dose fed study in healthy Chinese adult subjects [42]	2021	5	https://doi.org/10.5414/CP204002
				Long acting metformin vs metformin immediate release in patients with type 2 diabetes a systematic review [43]	2021	25	https://doi.org/10.3389/fphar.2021.669814
Ilze Elbere	Latvian Biomedical Research and Study Centre, Latvia	https://orcid.org/0000-0003-4381-885X	13	Metformin strongly affects gut microbiome composition in high fat diet induced type 2 diabetes mouse model of both sexes [44]	2021	49	https://doi.org/10.3389/fendo.2021.626359
				Baseline gut microbiome composition predicts metformin therapy short term efficacy in newly diagnosed type 2 diabetes patients [45]	2020	37	https://doi.org/10.1371/journal.pone.0241338
				Association of metformin administration with gut microbiome dysbiosis in healthy volunteers [46]	2018	98	https://doi.org/10.1371/journal.pone.0204317
Vivian Fonseca	Tulane University, The United States of America	https://orcid.org/0000-0002-3381-7151	103	Overview of metformin special focus on metformin extended release [47]	2012	56	https://doi.org/10.1517/14656566.2012.705829
				Efficacy tolerability and safety of a novel once daily extended release metformin in patients with type 2 diabetes [48]	2006	108	https://doi.org/10.2337/diacare.29.04.06.dc05-1967
				Diarrhea and metformin in a diabetic clinic [49]	1983	97	https://doi.org/10.2337/diacare.6.5.472
Janusz Gumprecht	Medical University of Silesia, Poland	https://orcid.org/0000-0001-7091-2118	32	Gastrointestinal adverse events of metformin treatment in patients with type 2 diabetes mellitus a systematic review and meta-analysis with meta regression of observational studies [50]	2024	6	https://doi.org/10.1186/s12902-024-01727-w
				Clinical trial probiotics in metformin intolerant patients with type 2 diabetes PROGASMET [51]	2023	6	https://doi.org/10.1016/j.biopha.2023.115650
				Gastrointestinal adverse events of metformin treatment in patients with type 2 diabetes mellitus a systematic review meta-analysis and meta regression of randomized controlled trials [52]	2022	48	https://doi.org/10.3389/fendo.2022.975912
Mitra Jelvehgari	Tabriz University of Medical Sciences, Iran	https://orcid.org/0000-0003-4247-9681	26	Expert design and optimization of a novel buccoadhesive blend film impregnated with metformin nanoparticles [53]	2020	5	https://doi.org/10.4155/tde-2020-0066
				Comparative study of in vitro release and mucoadhesivity of gastric compacts composed of multiple unit system/bilayered discs using direct compression of metformin hydrochloride [54]	2014	10	https://doi.org/10.5681/bi.2014.002
				Formulation and evaluation of in-vitro characterization of gastric-mucoadhesive microparticles/discs containing metformin hydrochloride [55]	2014	18	-
Ineta Kalniņa	Latvian Biomedical Research and Study Centre, Latvia	https://orcid.org/0000-0003-3462-3196	17	Metformin strongly affects gut microbiome composition in high fat diet induced type 2 diabetes mouse model of both sexes [44]	2021	49	https://doi.org/10.3389/fendo.2021.626359
				Association of metformin administration with gut microbiome dysbiosis in healthy volunteers [46]	2018	98	https://doi.org/10.1371/journal.pone.0204317
				Association of genetic variation in the organic cation transporters OCT1 OCT2 and multidrug and toxin extrusion 1 transporter protein genes with the gastrointestinal side effects and lower BMI in metformin treated type 2 diabetes patients [56]	2012	117	https://doi.org/10.1097/FPC.0b013e3283561666
Fatemeh Khonsari	Tabriz University of Medical Sciences, Iran	https://orcid.org/0000-0002-4651-5988	8	Formulation and evaluation of in vitro characterization of gastric mucoadhesive micro-particles discs containing metformin hydrochloride [55]	2014	18	-
				Comparative study of in vitro release and mucoadhesivity of gastric compacts composed of multiple unit system bilayered discs using direct compression of metformin hydrochloride [54]	2014	10	https://doi.org/10.5681/bi.2014.002
				Formulation and evaluation of in vitro characterization of gastric mucoadhesive micro-particles discs containing metformin hydrochloride [54]	2014	18	-
Jānis Kloviņš	Latvian Biomedical Research and Study Centre, Latvia	https://orcid.org/0000-0001-8362-5505	44	Metformin strongly affects gut microbiome composition in high fat diet induced type 2 diabetes mouse model of both sexes [44]	2021	49	https://doi.org/10.3389/fendo.2021.626359
				Association of metformin administration with gut microbiome dysbiosis in healthy volunteers [46]	2018	98	https://doi.org/10.1371/journal.pone.0204317
				Association of genetic variation in the organic cation transporters OCT1 OCT2 and multidrug and toxin extrusion 1 transporter protein genes with the gastrointestinal side effects and lower BMI in metformin treated type 2 diabetes patients [56]	2012	117	https://doi.org/10.1097/FPC.0b013e3283561666
Hanna Kwiendacz	Medical University of Silesia, Poland	https://orcid.org/0000-0002-1256-0414	13	Gastrointestinal adverse events of metformin treatment in patients with type 2 diabetes mellitus a systematic review and meta-analysis with meta regression of observational studies [50]	2024	6	https://doi.org/10.1186/s12902-024-01727-w
				Clinical trial probiotics in metformin intolerant patients with type 2 diabetes PROGASMET [51]	2023	6	https://doi.org/10.1016/j.biopha.2023.115650
				Gastrointestinal adverse events of metformin treatment in patients with type 2 diabetes mellitus a systematic review meta-analysis and meta regression of randomized controlled trials [52]	2022	48	https://doi.org/10.3389/fendo.2022.975912

Notes: “-” indicates no data available.

**Table 3:** Distribution of studies included in this bibliometric analysis by thematic area (*n* = 153).

Thematic Area	Number of Studies, *n* (%)	Topics Covered
Gastrointestinal effects	43 (28.1)	Diarrhea, nausea, abdominal discomfort, gastrointestinal intolerance, mechanisms of gastrointestinal adverse effects
Pharmaceutical formulations and delivery systems	13 (8.5)	Modified-release formulations, drug delivery systems, mucoadhesive systems, permeability enhancement strategies
Pharmacokinetics and pharmacodynamics	12 (7.8)	Drug absorption, bioavailability, dose–response relationship, transporters, drug metabolism
Gut microbiota	10 (6.5)	Microbiome composition, dysbiosis, microbiota–drug interaction, pharmacomicrobiomics
Clinical efficacy and glycemic control	7 (4.6)	Glycemic response, therapeutic effectiveness, glucose control, treatment outcomes
Treatment adherence and tolerability	2 (1.3)	Patient adherence, treatment discontinuation, tolerability, dose adjustment strategies
Molecular mechanisms and metabolic pathways	18 (11.8)	Cellular signaling pathways, transporter proteins, genetic polymorphisms, metabolic regulation
Comorbidities and associated diseases	16 (10.5)	Obesity, metabolic syndrome, cardiovascular disease, renal impairment
Combination therapy and pharmacological combinations	12 (7.8)	Co-administration with probiotics, antidiabetic agents, or supportive therapies
Safety and non-gastrointestinal adverse effects	10 (6.5)	Systemic adverse effects, metabolic disturbances, safety profile evaluation
Specific populations	10 (6.5)	Elderly patients, newly diagnosed individuals, metformin-intolerant patients, patients with genetic variability, sex-related differences

## Data Availability

The original contributions presented in this study are included in this article/Supplementary Materials. Further inquiries can be directed to the corresponding author.

## Supplementary Materials

The following supporting information can be downloaded at: https://www.mdpi.com/article/10.3390/medsci14060621/s1, Table S1. Complete search strategies used in Web of Science and Scopus.
