# Supplementary material for: Exploring Mechanisms and Therapeutic Strategies for Metformin Hydrochloride-Induced Diarrhea and Gastrointestinal Intolerance: A Bibliometric Analysis
Source: Med Sci (Basel). 2026 Sep 30;14(6):621. doi: 10.3390/medsci14060621 (PMC13706734; doi:10.3390/medsci14060621)
Supplement: Supplementary file 1 [file medsci-14-00621-s001.zip › medsci-4567240-supplementary.pdf]

SUPPLEMENTARY MATERIAL

Table S1. Complete search strategies used in Web of Science and Scopus.

Database	Search field	Full Boolean search string	Filters Applied	Records before filters	Records after filters	Search date
Web of Science	Topic	(metformin) AND (“gastrointestinal intolerance” OR diarrhea OR “gastrointestinal adverse effect*” OR “gastrointestinal side effect*” OR “digestive intolerance” OR “abdominal discomfort” OR “abdominal pain” OR “gastrointestinal tract”)	Article; Review Article	907	878	October 29, 2025
Scopus	TITLE-ABS-KEY	(metformin) AND (“gastrointestinal intolerance” OR diarrhea OR “gastrointestinal adverse effect*” OR “gastrointestinal side effect*” OR “digestive intolerance” OR “abdominal discomfort” OR “abdominal pain” OR “gastrointestinal tract”)	Article; Review	6091	5611	October 29, 2025
